# *trans*-Dibromidobis[diphen­yl(*p*-tol­yl)phosphine]palladium(II)

**DOI:** 10.1107/S1600536809045401

**Published:** 2009-11-14

**Authors:** Leo Kirsten, Gideon Steyl, Andreas Roodt

**Affiliations:** a Department of Chemistry, University of the Free State, PO Box 339, Bloemfontein 9300, South Africa

## Abstract

In the title compound, [PdBr_2_(C_19_H_17_P)_2_], the Pd^II^ ion resides on a centre of symmetry and is coordinated by two Br anions [Pd—Br = 2.4266 (2) Å] and two P-donor ligands [Pd—P = 2.3462 (5) Å] in a slightly distorted square-planar geometry [P—Pd—Br = 93.528 (12)°]. Weak inter­molecular C—H⋯Br hydrogen bonds link mol­ecules into chains extended in [1
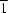
0].

## Related literature

For the isostructural compound, *trans*-[PdCl_2_{P((Ph)_2_(*p*-Tol))}_2_], in which the Pd centers are coordinated by Cl anions instead of Br, see: Steyl *et al.* (2006 ▶).
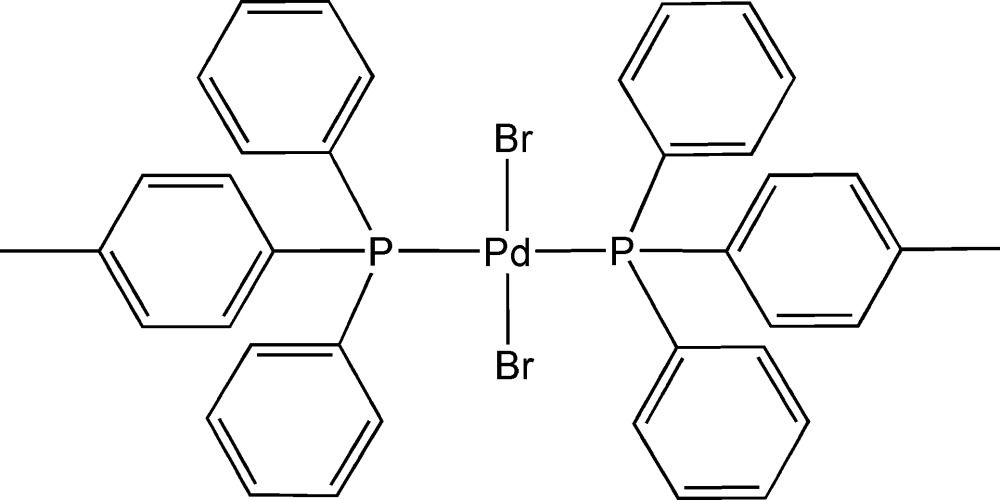


## Experimental

### 

#### Crystal data

[PdBr_2_(C_19_H_17_P)_2_]
*M* *_r_* = 818.81
Triclinic, 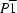

*a* = 10.0321 (4) Å
*b* = 10.0521 (4) Å
*c* = 10.2967 (4) Å
α = 70.876 (2)°
β = 68.288 (2)°
γ = 60.312 (2)°
*V* = 824.54 (6) Å^3^
*Z* = 1
Mo *K*α radiation
μ = 3.11 mm^−1^
*T* = 100 K
0.33 × 0.11 × 0.09 mm

#### Data collection

Bruker X8 APEXII 4K Kappa CCD diffractometer
Absorption correction: multi-scan (*SADABS*; Bruker, 1998 ▶) *T*_min_ = 0.427, *T*_max_ = 0.767
25915 measured reflections
3982 independent reflections
3621 reflections with *I* > 2σ(*I*)
*R*_int_ = 0.029

#### Refinement

*R*[*F* ^2^ > 2σ(*F* ^2^)] = 0.021
*wR*(*F* ^2^) = 0.051
*S* = 1.04
3982 reflections
196 parameters
H-atom parameters constrained
Δρ_max_ = 0.79 e Å^−3^
Δρ_min_ = −0.46 e Å^−3^

### 

Data collection: *APEX2* (Bruker, 2005 ▶); cell refinement: *SAINT-Plus* (Bruker, 2004 ▶); data reduction: *SAINT-Plus* and *XPREP* (Bruker, 2004 ▶); program(s) used to solve structure: *SHELXS97* (Sheldrick, 2008 ▶); program(s) used to refine structure: *SHELXL97* (Sheldrick, 2008 ▶); molecular graphics: *DIAMOND* (Brandenburg & Putz, 2006 ▶); software used to prepare material for publication: *SHELXL97*.

## Supplementary Material

Crystal structure: contains datablocks I, global. DOI: 10.1107/S1600536809045401/cv2640sup1.cif

Structure factors: contains datablocks I. DOI: 10.1107/S1600536809045401/cv2640Isup2.hkl

Additional supplementary materials:  crystallographic information; 3D view; checkCIF report

## Figures and Tables

**Table 1:** Hydrogen-bond geometry (Å, °)

*D*—H⋯*A*	*D*—H	H⋯*A*	*D*⋯*A*	*D*—H⋯*A*
C33—H33⋯Br^i^	0.93	2.88	3.7498 (19)	157
C22—H22⋯Br^ii^	0.93	2.71	3.501 (2)	144

Symmetry codes: (i) 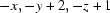; (ii) 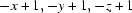.

**Fig. 1.**
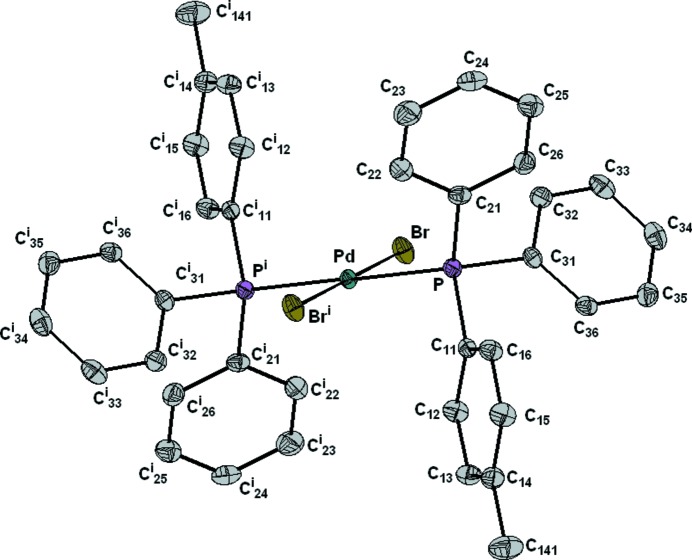
A sketch of the title compound showing the atomic numbering and 50% probability displacement ellipsoids [symmetry code: (i) 1 - x,1 - y,1 - z]. For the phenyl rings the first digit refers to ring number and the second digit to the atom in the ring. Hydrogen atoms have been omitted for clarity.

## supplementary crystallographic information

### Comment

This study is aimed at expanding the knowledge of *trans* square-planar palladium complexes containing phosphine donor ligands and bromido or chlorido anions as coordinating atoms.

The title compound is centrosymmetric with a slightly distorted-square-planar geometry, as seen by the *cis* angle P—Pd—Br of 93.528 (12)°. The packing of the title compound is stabilized by two weak intermolecular C—H···Br hydrogen bonds (Table 1).

The corresponding chloro complex (Steyl *et al.*, 2006) is iso-structural to the title complex when comparing the geometrical parameters as well as the crystallization mode. The RMS error of 0.061 Å also indicate the iso-structurality of the two complexes (the title complex superimposed with the corresponding dichloro-palladium complex (Steyl *et al.*, 2006) including the Pd, Cl(Br), P and first C atoms of the phenyl rings).

### Experimental

The title complex was synthesized by the addition of 2.2 equivalents of diphenyl(*p*-tolyl)phosphine (16 mg, 0.059 mmol) to [Pd(COD)Br_2_] (10 mg, 0.026 mmol) in 10 ml of dichloromethane while stirring for 5 minutes. Slow evaporation of the solvent resulted in orange crystals suitable for X-Ray diffraction (yield 71%, 16 mg).

### Refinement

All H atoms were positioned geometrically (C—H = 0.95 or 0.98 Å) and allowed to ride on their parent atoms, with *U*_iso_(H) = (1.2 or 1.5) times *U*_eq_ of the parent atom, respectively. The s.u.'s on all the Cell Axes and all the Cell Angles are equal as calculated from the unit cell determination.

### Crystal data

**Table:**

[PdBr_2_(C_19_H_17_P)_2_]	*Z* = 1
*M**_r_* = 818.81	*F*(000) = 408
Triclinic, *P*1	*D*_x_ = 1.649 Mg m^−^^3^
Hall symbol: -P 1	Mo *K*α radiation, λ = 0.71073 Å
*a* = 10.0321 (4) Å	Cell parameters from 6740 reflections
*b* = 10.0521 (4) Å	θ = 2.4–28.3°
*c* = 10.2967 (4) Å	µ = 3.11 mm^−^^1^
α = 70.876 (2)°	*T* = 100 K
β = 68.288 (2)°	Cuboid, orange
γ = 60.312 (2)°	0.33 × 0.11 × 0.09 mm
*V* = 824.54 (6) Å^3^	

### Data collection

**Table:**

Bruker X8 APEXII 4K Kappa CCD diffractometer	3982 independent reflections
Radiation source: fine-focus sealed tube	3621 reflections with *I* > 2σ(*I*)
graphite	*R*_int_ = 0.029
Detector resolution: 512 pixels mm^-1^	θ_max_ = 28.0°, θ_min_ = 2.4°
φ and ω scans	*h* = −13→13
Absorption correction: multi-scan (*SADABS*; Bruker, 1998)	*k* = −13→13
*T*_min_ = 0.427, *T*_max_ = 0.767	*l* = −13→13
25915 measured reflections	

### Refinement

**Table:**

Refinement on *F*^2^	Primary atom site location: structure-invariant direct methods
Least-squares matrix: full	Secondary atom site location: difference Fourier map
*R*[*F*^2^ > 2σ(*F*^2^)] = 0.021	Hydrogen site location: riding model
*wR*(*F*^2^) = 0.051	H-atom parameters constrained
*S* = 1.04	*w* = 1/[σ^2^(*F*_o_^2^) + (0.0199*P*)^2^ + 0.7898*P*] where *P* = (*F*_o_^2^ + 2*F*_c_^2^)/3
3982 reflections	(Δ/σ)_max_ = 0.001
196 parameters	Δρ_max_ = 0.79 e Å^−^^3^
0 restraints	Δρ_min_ = −0.46 e Å^−^^3^

### Special details

**Table:**

Geometry. All e.s.d.'s (except the e.s.d. in the dihedral angle between two l.s. planes) are estimated using the full covariance matrix. The cell e.s.d.'s are taken into account individually in the estimation of e.s.d.'s in distances, angles and torsion angles; correlations between e.s.d.'s in cell parameters are only used when they are defined by crystal symmetry. An approximate (isotropic) treatment of cell e.s.d.'s is used for estimating e.s.d.'s involving l.s. planes.
Refinement. Refinement of *F*^2^ against ALL reflections. The weighted *R*-factor *wR* and goodness of fit *S* are based on *F*^2^, conventional *R*-factors *R* are based on *F*, with *F* set to zero for negative *F*^2^. The threshold expression of *F*^2^ > σ(*F*^2^) is used only for calculating *R*-factors(gt) *etc*. and is not relevant to the choice of reflections for refinement. *R*-factors based on *F*^2^ are statistically about twice as large as those based on *F*, and *R*- factors based on ALL data will be even larger.

### Fractional atomic coordinates and isotropic or equivalent isotropic displacement parameters (Å^2^)

**Table:**

	*x*	*y*	*z*	*U*_iso_*/*U*_eq_	
Pd	0.5000	0.5000	0.5000	0.01341 (5)	
Br	0.39230 (2)	0.78196 (2)	0.45123 (2)	0.02035 (6)	
P	0.28802 (5)	0.49477 (5)	0.69762 (5)	0.01335 (9)	
C11	0.3682 (2)	0.3708 (2)	0.84911 (18)	0.0139 (3)	
C12	0.4892 (2)	0.3878 (2)	0.8690 (2)	0.0189 (4)	
H12	0.5284	0.4573	0.8035	0.023*	
C13	0.5507 (2)	0.3014 (2)	0.9859 (2)	0.0195 (4)	
H13	0.6301	0.3149	0.9986	0.023*	
C14	0.4963 (2)	0.1951 (2)	1.0847 (2)	0.0195 (4)	
C15	0.3776 (2)	0.1772 (2)	1.0628 (2)	0.0210 (4)	
H15	0.3408	0.1053	1.1267	0.025*	
C16	0.3131 (2)	0.2648 (2)	0.9470 (2)	0.0178 (4)	
H16	0.2327	0.2523	0.9351	0.021*	
C141	0.5660 (3)	0.1007 (3)	1.2101 (2)	0.0332 (5)	
H14A	0.5145	0.0339	1.2670	0.050*	
H14B	0.5504	0.1693	1.2662	0.050*	
H14C	0.6774	0.0386	1.1772	0.050*	
C21	0.1532 (2)	0.4290 (2)	0.68471 (19)	0.0160 (3)	
C22	0.2059 (2)	0.3178 (2)	0.6032 (2)	0.0235 (4)	
H22	0.3122	0.2743	0.5549	0.028*	
C23	0.1014 (3)	0.2714 (3)	0.5934 (2)	0.0281 (5)	
H23	0.1386	0.1952	0.5404	0.034*	
C24	−0.0579 (2)	0.3376 (2)	0.6621 (2)	0.0229 (4)	
H24	−0.1280	0.3071	0.6543	0.027*	
C25	−0.1120 (2)	0.4490 (2)	0.7422 (2)	0.0225 (4)	
H25	−0.2192	0.4945	0.7878	0.027*	
C26	−0.0077 (2)	0.4938 (2)	0.7553 (2)	0.0202 (4)	
H26	−0.0449	0.5673	0.8114	0.024*	
C31	0.1493 (2)	0.6787 (2)	0.75882 (19)	0.0144 (3)	
C32	0.0568 (2)	0.8003 (2)	0.6700 (2)	0.0181 (4)	
H32	0.0697	0.7872	0.5801	0.022*	
C33	−0.0535 (2)	0.9396 (2)	0.7151 (2)	0.0218 (4)	
H33	−0.1149	1.0196	0.6558	0.026*	
C34	−0.0728 (2)	0.9604 (2)	0.8489 (2)	0.0224 (4)	
H34	−0.1464	1.0545	0.8788	0.027*	
C35	0.0175 (2)	0.8410 (2)	0.9375 (2)	0.0202 (4)	
H35	0.0041	0.8547	1.0274	0.024*	
C36	0.1286 (2)	0.7006 (2)	0.89275 (19)	0.0160 (3)	
H36	0.1892	0.6208	0.9527	0.019*	

### Atomic displacement parameters (Å^2^)

**Table:**

	*U*^11^	*U*^22^	*U*^33^	*U*^12^	*U*^13^	*U*^23^
Pd	0.01211 (9)	0.01332 (9)	0.01158 (9)	−0.00500 (7)	−0.00154 (7)	−0.00073 (7)
Br	0.01631 (9)	0.01435 (9)	0.02261 (10)	−0.00509 (7)	−0.00119 (7)	−0.00071 (7)
P	0.0124 (2)	0.0147 (2)	0.0114 (2)	−0.00587 (17)	−0.00238 (16)	−0.00109 (16)
C11	0.0122 (8)	0.0143 (8)	0.0127 (8)	−0.0038 (7)	−0.0030 (6)	−0.0027 (6)
C12	0.0188 (9)	0.0186 (9)	0.0207 (9)	−0.0112 (7)	−0.0061 (7)	0.0014 (7)
C13	0.0189 (9)	0.0206 (9)	0.0229 (10)	−0.0094 (8)	−0.0088 (7)	−0.0030 (8)
C14	0.0199 (9)	0.0182 (9)	0.0178 (9)	−0.0057 (7)	−0.0071 (7)	−0.0016 (7)
C15	0.0210 (9)	0.0222 (10)	0.0191 (9)	−0.0130 (8)	−0.0051 (7)	0.0035 (7)
C16	0.0158 (8)	0.0197 (9)	0.0183 (9)	−0.0096 (7)	−0.0046 (7)	−0.0003 (7)
C141	0.0392 (13)	0.0362 (13)	0.0271 (11)	−0.0181 (11)	−0.0190 (10)	0.0065 (10)
C21	0.0177 (9)	0.0173 (9)	0.0138 (8)	−0.0090 (7)	−0.0068 (7)	0.0021 (7)
C22	0.0188 (9)	0.0258 (10)	0.0261 (10)	−0.0065 (8)	−0.0061 (8)	−0.0095 (8)
C23	0.0297 (11)	0.0280 (11)	0.0338 (12)	−0.0112 (9)	−0.0117 (9)	−0.0119 (9)
C24	0.0278 (10)	0.0268 (10)	0.0216 (10)	−0.0166 (9)	−0.0132 (8)	0.0028 (8)
C25	0.0198 (9)	0.0285 (10)	0.0198 (10)	−0.0140 (8)	−0.0048 (8)	0.0008 (8)
C26	0.0202 (9)	0.0249 (10)	0.0169 (9)	−0.0113 (8)	−0.0024 (7)	−0.0053 (8)
C31	0.0111 (8)	0.0160 (8)	0.0155 (8)	−0.0073 (7)	−0.0009 (6)	−0.0024 (7)
C32	0.0161 (8)	0.0214 (9)	0.0151 (9)	−0.0082 (7)	−0.0039 (7)	−0.0007 (7)
C33	0.0158 (9)	0.0179 (9)	0.0265 (10)	−0.0063 (7)	−0.0061 (8)	0.0015 (8)
C34	0.0168 (9)	0.0174 (9)	0.0306 (11)	−0.0067 (8)	−0.0014 (8)	−0.0079 (8)
C35	0.0194 (9)	0.0238 (10)	0.0213 (9)	−0.0115 (8)	−0.0015 (7)	−0.0087 (8)
C36	0.0142 (8)	0.0187 (9)	0.0169 (9)	−0.0095 (7)	−0.0041 (7)	−0.0012 (7)

### Geometric parameters (Å, °)

**Table:**

Pd—P	2.3462 (5)	C21—C26	1.399 (3)
Pd—P^i^	2.3462 (5)	C22—C23	1.387 (3)
Pd—Br^i^	2.4266 (2)	C22—H22	0.9300
Pd—Br	2.4266 (2)	C23—C24	1.384 (3)
P—C11	1.8150 (18)	C23—H23	0.9300
P—C31	1.8217 (18)	C24—C25	1.379 (3)
P—C21	1.8331 (19)	C24—H24	0.9300
C11—C16	1.387 (3)	C25—C26	1.388 (3)
C11—C12	1.399 (2)	C25—H25	0.9300
C12—C13	1.385 (3)	C26—H26	0.9300
C12—H12	0.9300	C31—C36	1.391 (3)
C13—C14	1.389 (3)	C31—C32	1.401 (3)
C13—H13	0.9300	C32—C33	1.383 (3)
C14—C15	1.392 (3)	C32—H32	0.9300
C14—C141	1.505 (3)	C33—C34	1.389 (3)
C15—C16	1.391 (3)	C33—H33	0.9300
C15—H15	0.9300	C34—C35	1.383 (3)
C16—H16	0.9300	C34—H34	0.9300
C141—H14A	0.9600	C35—C36	1.391 (3)
C141—H14B	0.9600	C35—H35	0.9300
C141—H14C	0.9600	C36—H36	0.9300
C21—C22	1.390 (3)		
			
P—Pd—P^i^	180.0	C22—C21—C26	118.53 (17)
P—Pd—Br^i^	86.472 (12)	C22—C21—P	121.58 (14)
P^i^—Pd—Br^i^	93.528 (12)	C26—C21—P	119.87 (14)
P—Pd—Br	93.528 (12)	C23—C22—C21	120.60 (19)
P^i^—Pd—Br	86.472 (12)	C23—C22—H22	119.7
Br^i^—Pd—Br	180.0	C21—C22—H22	119.7
C11—P—C31	103.51 (8)	C24—C23—C22	120.5 (2)
C11—P—C21	107.56 (8)	C24—C23—H23	119.8
C31—P—C21	101.59 (8)	C22—C23—H23	119.8
C11—P—Pd	108.36 (6)	C25—C24—C23	119.51 (19)
C31—P—Pd	116.57 (6)	C25—C24—H24	120.2
C21—P—Pd	118.01 (6)	C23—C24—H24	120.2
C16—C11—C12	118.91 (16)	C24—C25—C26	120.46 (19)
C16—C11—P	123.69 (14)	C24—C25—H25	119.8
C12—C11—P	117.36 (14)	C26—C25—H25	119.8
C13—C12—C11	120.18 (17)	C25—C26—C21	120.41 (18)
C13—C12—H12	119.9	C25—C26—H26	119.8
C11—C12—H12	119.9	C21—C26—H26	119.8
C12—C13—C14	121.38 (17)	C36—C31—C32	118.96 (17)
C12—C13—H13	119.3	C36—C31—P	122.06 (14)
C14—C13—H13	119.3	C32—C31—P	118.94 (14)
C13—C14—C15	118.01 (17)	C33—C32—C31	120.42 (18)
C13—C14—C141	120.66 (18)	C33—C32—H32	119.8
C15—C14—C141	121.32 (18)	C31—C32—H32	119.8
C16—C15—C14	121.24 (18)	C32—C33—C34	120.13 (18)
C16—C15—H15	119.4	C32—C33—H33	119.9
C14—C15—H15	119.4	C34—C33—H33	119.9
C11—C16—C15	120.26 (17)	C35—C34—C33	119.91 (18)
C11—C16—H16	119.9	C35—C34—H34	120.0
C15—C16—H16	119.9	C33—C34—H34	120.0
C14—C141—H14A	109.5	C34—C35—C36	120.19 (18)
C14—C141—H14B	109.5	C34—C35—H35	119.9
H14A—C141—H14B	109.5	C36—C35—H35	119.9
C14—C141—H14C	109.5	C31—C36—C35	120.38 (17)
H14A—C141—H14C	109.5	C31—C36—H36	119.8
H14B—C141—H14C	109.5	C35—C36—H36	119.8
			
Br^i^—Pd—P—C11	54.51 (6)	C11—P—C21—C26	90.47 (16)
Br—Pd—P—C11	−125.49 (6)	C31—P—C21—C26	−17.89 (17)
Br^i^—Pd—P—C31	170.70 (7)	Pd—P—C21—C26	−146.67 (13)
Br—Pd—P—C31	−9.30 (7)	C26—C21—C22—C23	−0.6 (3)
Br^i^—Pd—P—C21	−67.94 (7)	P—C21—C22—C23	−179.11 (16)
Br—Pd—P—C21	112.06 (7)	C21—C22—C23—C24	1.5 (3)
C31—P—C11—C16	97.53 (16)	C22—C23—C24—C25	−0.9 (3)
C21—P—C11—C16	−9.49 (18)	C23—C24—C25—C26	−0.6 (3)
Pd—P—C11—C16	−138.11 (14)	C24—C25—C26—C21	1.5 (3)
C31—P—C11—C12	−80.59 (15)	C22—C21—C26—C25	−0.9 (3)
C21—P—C11—C12	172.39 (14)	P—C21—C26—C25	177.67 (15)
Pd—P—C11—C12	43.78 (15)	C11—P—C31—C36	1.98 (17)
C16—C11—C12—C13	−1.0 (3)	C21—P—C31—C36	113.45 (15)
P—C11—C12—C13	177.24 (15)	Pd—P—C31—C36	−116.86 (14)
C11—C12—C13—C14	0.9 (3)	C11—P—C31—C32	−175.85 (14)
C12—C13—C14—C15	0.1 (3)	C21—P—C31—C32	−64.39 (16)
C12—C13—C14—C141	179.2 (2)	Pd—P—C31—C32	65.30 (15)
C13—C14—C15—C16	−1.1 (3)	C36—C31—C32—C33	−0.1 (3)
C141—C14—C15—C16	179.83 (19)	P—C31—C32—C33	177.80 (14)
C12—C11—C16—C15	0.0 (3)	C31—C32—C33—C34	0.4 (3)
P—C11—C16—C15	−178.07 (15)	C32—C33—C34—C35	−0.6 (3)
C14—C15—C16—C11	1.0 (3)	C33—C34—C35—C36	0.5 (3)
C11—P—C21—C22	−91.04 (17)	C32—C31—C36—C35	0.0 (3)
C31—P—C21—C22	160.60 (16)	P—C31—C36—C35	−177.80 (14)
Pd—P—C21—C22	31.82 (18)	C34—C35—C36—C31	−0.2 (3)

Symmetry codes: (i) −*x*+1, −*y*+1, −*z*+1.

### Hydrogen-bond geometry (Å, °)

**Table:**

*D*—H···*A*	*D*—H	H···*A*	*D*···*A*	*D*—H···*A*
C32—H32···Br	0.93	2.99	3.2380 (18)	97
C33—H33···Br^ii^	0.93	2.88	3.7498 (19)	157
C22—H22···Br^i^	0.93	2.71	3.501 (2)	144

Symmetry codes: (ii) −*x*, −*y*+2, −*z*+1; (i) −*x*+1, −*y*+1, −*z*+1.

## References

[bb1] Brandenburg, K. & Putz, H. (2006). *DIAMOND*. Crystal Impact GbR, Bonn, Germany.

[bb2] Bruker (1998). *SADABS*. Bruker AXS Inc., Madison, Wisconsin, USA.

[bb3] Bruker (2004). *SAINT-Plus* (including *XPREP*). Bruker AXS Inc., Madison, Wisconsin, USA.

[bb4] Bruker (2005). *APEX2*. Bruker AXS Inc., Madison, Wisconsin, USA.

[bb5] Sheldrick, G. M. (2008). *Acta Cryst* A**64**, 112–122. 10.1107/S0108767307043930 18156677

[bb6] Steyl, G., Kirsten, L. & Roodt, A. (2006). *Acta Cryst.* E**62**, m1705–m1707.

